# 

**Published:** 1872-03

**Authors:** 


### ANNUAL MEETING OF THE GEORGIA MEDICAL ASSOCIATION.

The Annual Meeting of the Georgia Medical Association will take place at Columbus, on the second Wednesday of April—the 10th prox.

It is expected that arrangements will be made with the various railroads to pass the members attending the meeting upon the payment of half fare. A full attendance of the members is expected.

GEO. M. McDOWELL, M.D., President.

S. H. Stout, M.D., Permanent Secretary.

COMMITTEE OF ARRANGEMENTS.

E. J. Kirkscey, M.D., F. A. Stanford, M.D.,
V. H. Taliaferro, M.D., T. F. Brewster, M.D.,
S. A Billings, M. D.

